# Challenges and strategies in building a foundational digital health data integration ecosystem: a systematic review and thematic synthesis

**DOI:** 10.3389/frhs.2025.1600689

**Published:** 2025-06-20

**Authors:** Radha Ambalavanan, R Sterling Snead, Julia Marczika, Gideon Towett, Alex Malioukis, Mercy Mbogori-Kairichi

**Affiliations:** Research Department, The Self Research Institute, Broken Arrow, OK, United States

**Keywords:** patient-centered care, healthcare data integration, systematic review, interoperability, genomic data, thematic synthesis, digital health ecosystem

## Abstract

**Background:**

Chronic conditions require robust healthcare data integration to support personalized care, real-time decision-making, and secure information exchange. However, fragmented data ecosystems disrupt interoperability, complicate patient-centered care (PCC), and present challenges for incorporating genomic data into clinical workflows.

**Objective:**

This systematic review with thematic synthesis aims to identify key challenges and synthesize existing strategies from the literature to inform the development of a foundational digital health data integration ecosystem.

**Methods:**

Following PRISMA guidelines, we systematically screened literature across multiple databases. A thematic synthesis approach was used to categorize findings into three primary themes: interoperability, PCC, and genomic data integration.

**Results:**

A total of 161 studies were included. Key challenges identified include semantic misalignment across commonly used healthcare standards such as HL7 FHIR and SNOMED CT, limited cross-system data exchange, inadequate patient engagement features in EHRs, and concerns regarding the security and clinical utility of genomic data. Strategies described across the literature include ontology-based interoperability models, AI-supported PCC frameworks, and blockchain-enabled genomic data governance.

**Conclusion:**

By analyzing current methodologies, research gaps, and implementation challenges, this review offers an evidence-based foundation to guide future advancements in healthcare data integration. It supports the development of scalable, privacy-preserving, and ethically governed data-sharing infrastructures that enable personalized medicine and real-time clinical interventions.

**Systematic Review Registration:**

https://osf.io/c2xvw.

## 1 Introduction

Chronic conditions, as defined by the World Health Organization (WHO), require sustained, long-term management and present multifaceted challenges across multiple health domains. These conditions encompass cardiovascular diseases, diabetes mellitus, asthma, HIV/AIDS, psychiatric disorders, sensory impairments, musculoskeletal disorders, and oncology-related diseases. Addressing this diverse spectrum necessitates a multidisciplinary approach prioritizing patient engagement, effective monitoring, and access to pharmacotherapeutics (1, 2). These challenges underscore the importance of integrated data systems to support PCC and personalized healthcare delivery.

Despite advancements in healthcare, fragmented systems often hinder collaboration and impair both research and care delivery. Inefficiencies, delays, and inadequate patient outcomes arise from the lack of seamless data integration (3). Interoperability remains a cornerstone of modern healthcare systems, requiring well-defined strategies for structuring, managing, and securely exchanging diverse health data. PCC, a foundational principle in modern healthcare, emphasizes empowerment, active participation, and collaboration among care team members, including patients, families, and providers. Nevertheless, PCC strategies require robust data systems to ensure effective care coordination and personalized interventions. As noted by Wagner et al. (4), coordinated care enhances collaboration and patient outcomes, a principle further supported by the Agency for Healthcare Research and Quality (AHRQ), which highlights metrics for capturing PCC strategies (5). In modern healthcare frameworks, PCC has become a central focus, strengthening trust, satisfaction, and communication (6).

While the adoption of EHRs has revolutionized data management, challenges such as data interoperability, security, and seamless integration of diverse data types persist. EHR-linked biobanks and genomic research initiatives have demonstrated the potential of combining phenotypic and genetic data to inform precision medicine. However, this potential remains limited without standardized approaches, these efforts face scalability limitations that hinder their broader adoption. Interoperability is particularly essential for chronic disease management, where effective data sharing ensures timely, high-quality healthcare. Without it, fragmented systems lead to redundant efforts and delayed treatments, ultimately affecting patient outcomes (7–10).

The transition from paper-based records to EHRs began in the early 1990s, driven by technological advancements and advocacy from the Institute of Medicine (11). While EHR adoption has accelerated globally, especially with initiatives like the Meaningful Use (MU) program in the United States, interoperability challenges remain a significant barrier to fully realizing their potential in modern healthcare (12, 13). Over the past 25 years, EHR adoption among office-based physician practices has seen significant growth. According to Health IT data (14), EHR adoption among office-based physician practices in the United States has risen from 18 percent in 2001 to 78 percent in 2013 for any EHR and from 72 percent in 2019 to 78 percent in 2021 for Certified EHR, reflecting the ongoing digital transformation in healthcare.

In their seminal work, the Institute of Medicine (IOM) (15) outlined Six Aims in Crossing the Quality Chasm, aiming to enhance healthcare delivery by improving safety, effectiveness, patient-centeredness, timeliness, efficiency, and equity—essential factors for delivering high-quality care, particularly for chronic conditions. These aims address fragmented data systems and emphasize integrated, patient-centered healthcare. The COVID-19 pandemic further underscored the need for comprehensive data integration strategies that enable continuity of care and informed decision-making during crises (16).

EHRs significantly streamline healthcare by centralizing patient data, improving accessibility for providers, and enabling direct patient access to medical records. Features such as secure communication, appointment scheduling, test result viewing, and medication management enhance patient convenience and engagement, fostering empowerment and collaboration (12, 17, 18). Although progress is evident, significant barriers remain in standardizing data interoperability, security, and genomic data integration. Overcoming these challenges is essential for enhancing personalized care and optimizing clinical decision-making.

Several studies have explored healthcare data integration frameworks, focusing on interoperability, PCC, and genomic data integration. Prior reviews highlight key challenges such as inconsistent terminology mapping, lack of standardized data-sharing models, and gaps in patient-centric engagement strategies. However, a comprehensive thematic synthesis of these challenges and potential solutions remains lacking. This systematic review addresses this gap by synthesizing existing evidence to bridge knowledge gaps and inform future research.

In the context of this review, interoperability denotes the ability of different health information systems to access, exchange, and meaningfully interpret shared data across various healthcare domains and organizational settings. PCC refers to a healthcare approach that emphasizes active patient engagement, shared decision-making, and individualized care planning. Genomic data integration involves incorporating genetic and molecular information into clinical workflows to support personalized treatment strategies. A digital health data ecosystem encompasses the interconnected infrastructure through which clinical, patient-generated, and genomic data are collected, shared, and utilized to enable real-time, data-driven healthcare.

Despite ongoing advancements in EHR adoption and interoperability frameworks, significant challenges remain in integrating diverse health data sources, ensuring seamless PCC, and leveraging genomic data for precision medicine. A structured, evidence-based synthesis is essential to identify barriers, evaluate existing methodologies, and propose best practices for improving healthcare data integration. To address these challenges, this systematic review follows PRISMA guidelines and employs a thematic synthesis approach to analyze studies on interoperability, PCC, and genomic data integration. The research framework is systematically structured using the PICOS (Population, Intervention, Comparator, Outcome, and Study Design) approach, which is detailed in the materials and methods section.

## 2 Materials and methods

### 2.1 Study design

This study followed a systematic review methodology, adhering to the Preferred Reporting Items for Systematic Reviews and Meta-Analyses (PRISMA) (19) guidelines to ensure transparency and reproducibility, see Supplementary File S1. Given the heterogeneity of study designs and outcome measures, a thematic synthesis approach was employed to identify patterns and themes related to interoperability, PCC, and genomic data integration. The systematic review aimed to categorize existing research gaps and methodologies, focusing on healthcare data interoperability. This systematic review was prospectively registered with the Open Science Framework (OSF): https://osf.io/c2xvw.

### 2.2 Literature search strategy

To capture a comprehensive set of studies, the literature search included multiple databases and supplementary sources, with studies identified up to March 15, 2024. The primary databases used were PubMed, which focuses on biomedical and life sciences literature; MEDLINE, which covers health-related literature; and Scopus, which provides broad scientific research coverage. Since MEDLINE and PubMed have significant overlap, they were treated as a single database to avoid redundancy in the search process.

In addition to these primary databases, Google Scholar and the WHO repository were included as supplementary sources to capture gray literature and studies not indexed in the primary databases. To mitigate publication bias and ensure inclusivity, the search strategy also encompassed online books, conference abstracts, seminars, and reference publications. Gray literature was selectively included based on relevance, credibility, and methodological rigor, with preference given to peer-reviewed reports, government publications, and institutional white papers. Conference abstracts were considered only if they provided empirical data relevant to interoperability, PCC, or genomic data integration. To broaden the scope, the reference lists of selected articles were manually screened to identify additional relevant studies.

### 2.3 Search syntax strategy

The search strategy focused on key concepts such as “Electronic Health Record,” “Genomic Data,” “Ontology Integration,” “Chronic Conditions,” “Patient-Centered Care,” “Interoperability,” and “Healthcare Quality.” Boolean operators (AND, OR, NOT) and advanced search techniques (quotation marks, wildcard characters, parentheses) refined the search to ensure comprehensive coverage of related terms and variations. The resulting search string included combinations such as (“Electronic Health Record” OR “Genomic Data” OR “Patient-Centered Care” OR “Interoperability”) AND (“Ontology Integration” OR “Healthcare Quality”). The complete search syntax is provided in the Supplementary File S2.

### 2.4 Article screening and selection

Articles potentially relevant to the study were initially screened based on their titles and imported into Rayyan, a free web-based application designed to support article screening in systematic reviews for efficient management and blinded screening by independent reviewers. Further selection involved abstract and full-text reviews, applying Rayyan's screening tools (20) to eliminate duplicates and filter studies based on predefined inclusion and exclusion criteria, see Supplementary File S3, where a detailed table outlines the selection criteria.

While quantitative studies were not excluded *a priori*, they were only retained if they contributed conceptually relevant insights to thematic coding and qualitative synthesis. Purely quantitative studies focusing solely on statistical outcomes without conceptual or narrative interpretation were excluded, as they did not align with the review's thematic objectives. This approach ensured that the selected studies provided rich contextual information essential for evaluating healthcare data integration frameworks. To ensure alignment with study objectives, priority was given to studies involving healthcare providers, clinical workflows, and patient data analysis. Studies that exclusively discussed theoretical ontology models, generalized genomic concepts, or computational frameworks without direct relevance to EHRs, PCC, or interoperability were excluded.

A structured selection process was applied using the PICOS framework to define the review focus and inclusion criteria. This process supported systematic study selection aligned with the thematic structure of the review, as outlined in Table 1.

**Table 1:** Research question framework (PICOS).

PICOS element	Description in the context of this review
Population (P)	Healthcare systems handling chronic disease management and clinical decision-making through integrated use of EHRs, PGHD, and genomic data.
Intervention (I)	Use of interoperability frameworks (e.g., HL7 FHIR), PCC strategies, and genomic data integration models (e.g., SSI, blockchain-based governance).
Comparator (C)	Studies comparing conventional vs. advanced integration strategies, or evaluating the impact of structured vs. unstructured health data frameworks.
Outcome (O)	Enhanced interoperability, improved patient engagement, better chronic disease management, secure genomic data use, and more efficient, personalized care delivery.
Study design (S)	Systematic review using thematic synthesis, analyzing qualitative studies on healthcare data integration frameworks and technologies.

Following these criteria, titles were first assessed for relevance, followed by abstract reviews and a comprehensive review of full-text articles. Only studies meeting the predefined criteria were included, ensuring the integrity and validity of findings. A curated articles database supported this process; see Supplementary File S4—Sheet 1.

### 2.5 Data extraction and thematic categorization

The systematic review identified 161 relevant studies, analyzed using a structured thematic synthesis approach. The studies were categorized into three main themes: (i) Interoperability, focusing on challenges and strategies for integrating healthcare data across platforms; (ii) PCC, emphasizing patient engagement in data-driven care; and (iii) Genomic Data Integration, exploring methods for incorporating genomic information into clinical decision-making.

Relevant data were extracted manually using a structured template developed by the review team. Key variables included study design, sample size, methodology, bias level, quality rating, thematic category, and key findings. Each study was assessed using predefined criteria evaluating research aims, data collection methods, and potential limitations to ensure methodological rigor. Bias levels were categorized as low, moderate, or high based on structured criteria similar to ROBIS (Risk of Bias in Systematic Reviews) for systematic reviews and JBI (Joanna Briggs Institute) Critical Appraisal Checklists for qualitative studies.

The initial extraction was performed by the first author (RA), and independently reviewed and validated by the second reviewer (RSS) and third reviewer (JM). Discrepancies were resolved through consensus. Data were extracted and organized using structured spreadsheets. The categorization and quality assessment process are detailed in Supplementary File S5—Sheet 1. A comprehensive summary of extracted data is presented in Table 2, detailed thematic classification and key findings were recorded in Supplementary Files S5—Sheets 1 and 2.

**Table 2:** Key findings from systematic review and thematic synthesis.

Main theme	Challenges in healthcare data integration	Clinical implications	Proposed solutions & best practices
Interoperability	Fragmented data exchange due to lack of standardized protocols.	Delays in data access and decision-making.	Promote universal adoption of HL7 FHIR, SNOMED CT, and LOINC.
	Inconsistent adoption of HL7 FHIR, SNOMED CT, and LOINC across healthcare systems.	Incomplete or inconsistent patient records.	Develop ontology-based models for semantic alignment.
		Limited system scalability.	Standardize APIs for seamless platform exchange.
Patient-centered care (PCC)	Poor integration of PGHD into EHRs.	Reduced personalization of care.	Develop AI-driven platforms with real-time PGHD tracking.
	Lack of user-friendly tools for patient engagement.	Limited engagement in treatment decisions.	Embed CDS tools in EHRs using NLP and feedback loops.
		Lack of real-time feedback.	
Genomic data integration	Ethical and regulatory concerns.	Data privacy risks and consent violations.	Use federated learning for decentralized genomic analysis.
	Consent complexity and cross-border data governance.	Barriers to genomic medicine implementation.	Integrate genomic standards (OMOP-CDM, GA4GH) with EHRs.
	High computational cost of AI/genomics integration.	Inaccurate predictions due to processing gaps.	Apply privacy-preserving technologies like blockchain and ZKPs.

### 2.6 Thematic coding and reliability

Data extraction followed a structured thematic synthesis approach by three independent reviewers. Articles were systematically analyzed to highlight key excerpts, assign initial codes, and categorize findings under interoperability, PCC, and genomic data integration. Thematic coding was iteratively refined to capture emerging patterns. Themes were cross-validated through consensus discussions to ensure reliability. Discrepancies were resolved through deliberation, minimizing subjectivity. Annotation details, including coding and methodological assessment, were recorded in Supplementary File S5—Sheet 2. No statistical inter-rater reliability metric was calculated, as coding was consensus-based, with decisions refined iteratively.

### 2.7 Risk of bias and quality assessment

The risk of bias was qualitatively assessed based on study methodology, sample representativeness, and reporting transparency. A structured qualitative assessment adapted from conventional systematic review practices was implemented. Rather than using a formal checklist like ROBIS or JBI, the review team used predefined criteria to evaluate clarity in data collection methods, alignment with study objectives, and depth of analytical insight. Studies were categorized as having a low, moderate, or high risk of bias based on these criteria. The complete quality assessment details are provided in Supplementary File S5—Sheet 1.

### 2.8 Data synthesis approach

Due to the methodological diversity of included studies, a meta-analysis was not conducted. The heterogeneity in study designs, sample populations, and measured outcomes made effect-size comparisons infeasible. Studies encompassed qualitative case studies and observational studies, which vary significantly in analytical approaches. Instead, a thematic synthesis approach was adopted to systematically extract recurring patterns and research gaps, ensuring a structured and comprehensive synthesis of findings while maintaining methodological rigor.

## 3 Results

### 3.1 PRISMA flow diagram

**Figure 1:**
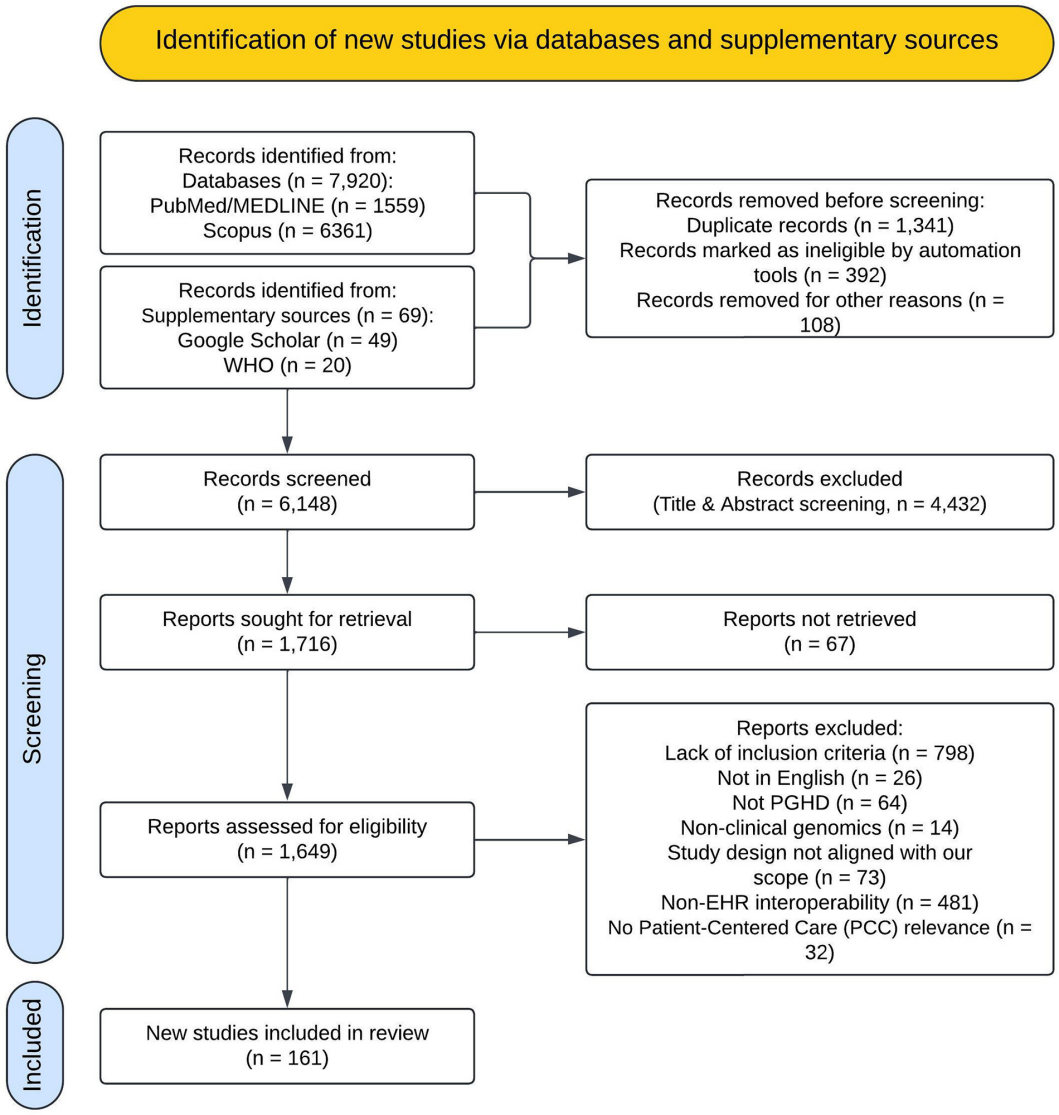
A visual representation illustrating the study selection process according to the preferred reporting items for systematic reviews and meta-analyses (PRISMA) guidelines. PCC, patient-centered care; EHR, electronic health record; PGHD, patient-generated health data.

The article selection process followed the PRISMA 2020 guidelines, ensuring a systematic and transparent approach to identifying, screening, and including studies. The initial search yielded 7,989 records from specified databases and supplementary sources such as Google Scholar and the WHO repository. After removing duplicates using Rayyan software, 6,148 unique records remained. Screening based on predefined inclusion and exclusion criteria resulted in 1,649 articles assessed for eligibility. Following thorough evaluation, 161 studies were included in the systematic review. Figure 1 (PRISMA Flow Diagram) visually represents the article selection process, highlighting the methodology's rigor and transparency.

### 3.2 Key themes from systematic review and thematic synthesis

The systematic review identified 161 relevant studies, which were analyzed to uncover recurring themes in healthcare data integration. The findings were categorized into three overarching themes: interoperability, PCC, and genomic data integration.

Interoperability challenges stem from fragmented data-sharing mechanisms, inconsistent adoption of interoperability standards such as Health Level Seven Fast Healthcare Interoperability Resources (HL7 FHIR), Systematized Nomenclature of Medicine Clinical Terms (SNOMED CT), and Logical Observation Identifiers Names and Codes (LOINC), as well as limitations in application programming interfaces (APIs) that restrict seamless healthcare system integration. These inconsistencies lead to inefficiencies in data exchange, making it difficult to achieve interoperability across different health information technology infrastructures.

Insights from PCC highlight barriers to patient engagement, including limited integration of patient-generated health data (PGHD) into EHRs, lack of real-time feedback mechanisms, and usability constraints in digital health platforms. The absence of structured patient engagement mechanisms reduces the effectiveness of PCC strategies, impacting treatment adherence and personalized care delivery.

Genomic data integration presents challenges related to privacy, security, and the computational burden of incorporating phenotypic-genotypic relationships into clinical workflows. Ethical concerns regarding data sharing, regulatory compliance, and the complexity of integrating genomic information within EHR systems further hinder the adoption of genomics-driven precision medicine.

These findings emphasize the pressing need for standardized frameworks, AI-driven data harmonization techniques, and security-enhanced genomic data models to support precision medicine and patient-centered interventions. Table 2 presents a structured summary of the key findings, their clinical implications, and proposed solutions to address these challenges. A more detailed breakdown of study annotations and thematic classifications is available in Supplementary Files S5.

Among the 161 included studies, the most common study designs were descriptive (*n* = 54), followed by qualitative studies (*n* = 15), and a range of implementation, observational, mixed-methods, and exploratory approaches (*n* = 92). The majority of studies (*n* = 150) were assessed to have low risk of bias, while a small number were rated as moderate or contained narrative descriptions of potential selection or reporting bias. In terms of thematic representation, interoperability was the most prevalent focus, appearing in 149 studies either independently or in combination with other concepts. Patient-centered care was identified in 48 studies, while genomic data integration featured in 14. These patterns are detailed in Table 2 and Supplementary Files S5—Sheets 1 and 2 were used to guide the thematic synthesis.

### 3.3 Challenges in healthcare data integration

The findings from this systematic review reveal persistent challenges in healthcare data interoperability, PCC implementation, and genomic data integration, emphasizing the need for standardized solutions.

Interoperability challenges stem from fragmented data-sharing mechanisms, inconsistent adoption of HL7 FHIR, SNOMED CT, and LOINC, and API standardization limitations. Studies included in this review indicate that semantic misalignment remains a key barrier, leading to inefficiencies in clinical workflows and delays in healthcare decision-making. The lack of unified interoperability frameworks across institutions and regions further complicates data exchange. Moreover, the absence of ontology-driven integration models prevents seamless semantic consistency, restricting the scalability and adaptability of health IT systems.

PCC implementation challenges identified in this review highlight limited integration of PGHD into EHR systems, usability constraints in digital health platforms, and a lack of structured real-time patient feedback mechanisms. Although various mobile health applications and patient portals have been developed to facilitate engagement, their integration into existing EHRs remains inconsistent, reducing their effectiveness in chronic disease management. Compounding these challenges, studies indicate that personalized care adjustments are often delayed due to the absence of dynamic, real-time data exchange between patients and providers. These limitations underscore the need for enhanced AI-driven automation and interoperability solutions to optimize patient engagement strategies.

Genomic data integration challenges primarily relate to data privacy, regulatory compliance, and computational inefficiencies. Several studies in this review point to concerns over data security and the complexities of aligning genomic datasets with phenotypic and clinical information. Ethical considerations, including patient consent models, risks of genetic discrimination, and varying international data-sharing regulations such as the Health Insurance Portability and Accountability Act (HIPAA) and the General Data Protection Regulation (GDPR), further complicate the adoption of genomics-driven precision medicine. Another significant barrier is the computational inefficiency in processing large-scale genomic datasets using AI-driven approaches, which limits the real-time clinical application of genomic insights.

The insights gathered from this review reinforce the need for structured, ontology-based frameworks, AI-driven data harmonization, and enhanced security measures to address these critical challenges and advance the integration of interoperability, PCC, and genomic data in healthcare systems.

### 3.4 Proposed solutions and best practices

Addressing the challenges identified in healthcare data integration requires structured interventions that enhance interoperability, PCC, and genomic data integration. Based on the findings from this systematic review, solutions must focus on standardization, technological advancements, and regulatory alignment to achieve seamless data exchange, improved patient engagement, and secure genomic data utilization.

#### 3.4.1 Enhancing interoperability through standardization and ontology integration

The fragmented adoption of interoperability standards across healthcare systems presents significant barriers to effective data exchange. To address these challenges, the widespread implementation of standards such as HL7 FHIR, SNOMED CT, and LOINC should be prioritized to promote semantic consistency and data harmonization. The integration of ontology-based frameworks can further enhance data standardization by enabling automated reasoning and supporting advanced decision-support tools. In parallel, the development and deployment of standardized APIs can improve cross-platform interoperability, thereby reducing workflow inefficiencies. To ensure widespread adoption, regulatory policies must reinforce interoperability mandates and promote universal compliance across healthcare systems.

#### 3.4.2 Advancing patient-centered care through digital health solutions

To overcome barriers in patient engagement and PCC implementation, healthcare systems should adopt AI-driven digital health platforms that offer real-time feedback mechanisms and decision-support tools. Mobile-first applications with intuitive user interfaces can enhance PGHD integration within EHR workflows, ensuring patients actively participate in their care. Embedding NLP-based decision-support tools can further personalize treatment plans by leveraging real-time patient data analytics.

#### 3.4.3 Strengthening data security and ethical governance in genomic data integration

Given the increasing role of genomics in precision medicine, ensuring data security and regulatory compliance is critical. Secure encryption models and decentralized consent frameworks can safeguard genomic data privacy while enabling responsible data sharing. Aligning genomic data standards such as the Observational Medical Outcomes Partnership Common Data Model (OMOP-CDM) and the Global Alliance for Genomics and Health (GA4GH) framework with clinical ontologies can facilitate better integration into electronic health record (EHR) systems. Federated learning techniques can allow AI-driven insights without centralizing sensitive patient data, enhancing both security and scalability.

By implementing these standardized interoperability frameworks, AI-driven PCC strategies, and secure genomic data integration practices, healthcare systems can move toward scalable, patient-centered, and ethically governed data ecosystems that support precision medicine and chronic disease management.

While many interoperability studies proposed technically sound frameworks, several lacked validations in real-world clinical settings, which limited their usefulness in actual healthcare environments. Studies on PCC were often grounded in strong conceptual frameworks but varied widely in how they measured actual patient engagement, often relying on provider-reported data rather than direct patient input. Genomic integration studies, though methodologically robust, frequently assumed infrastructural readiness and data standardization that may not reflect current realities across diverse healthcare systems.

### 3.5 Key findings and research gaps in healthcare data integration

This review identifies critical gaps in healthcare data standardization, PCC strategies, and genomic data security measures. While existing interoperability solutions aim to enhance healthcare data exchange, their implementation remains fragmented, resulting in semantic inconsistencies and scalability limitations across institutions. The findings underscore the necessity of structured data exchange models, the integration of standardized terminologies, and enhanced regulatory alignment to facilitate seamless healthcare data management.

Several emerging trends were observed, including the increasing use of ontology-driven data models to standardize clinical concepts and improve cross-platform interoperability. Self-Sovereign Identity (SSI) is gaining attention as a privacy-preserving approach that enables patients to retain control over their health information, genomic data, and digital identities. SSI frameworks could address data ownership concerns, ensuring secure authentication and cross-border data exchange without reliance on centralized authorities. The integration of genomic data within EHR systems has also gained momentum; yet privacy concerns, ethical considerations, and the lack of harmonized regulatory frameworks continue to hinder widespread adoption. Research findings also indicate that institutional-level interoperability frameworks are often more developed than national-scale initiatives, highlighting disparities in adoption rates and governance models.

Despite advancements in health IT, research gaps persist. Many existing interoperability frameworks lack the adaptability required for dynamic healthcare environments, limiting their effectiveness in real-world applications. The handling of Directly Identifiable Information (DII) and Indirectly Identifiable Information (III) presents additional challenges, as AI-driven analytics and genomic data integration raise concerns about re-identification risks, data misuse, and compliance with privacy regulations such as GDPR and HIPAA. Although AI-driven predictive analytics, blockchain for secure data exchange, and graph-based databases are promising, their adoption remains limited due to regulatory, computational, and technical constraints. Future research should focus on validating these technologies through empirical studies, addressing cross-border regulatory challenges, and developing scalable, flexible infrastructures to support real-world healthcare applications.

## 4 Discussion

### 4.1 Interoperability and data exchange limitations

Despite advancements in healthcare data integration, persistent gaps in semantic consistency, interoperability across platforms, and data standardization hinder effective healthcare communication and decision-making. Semantic inconsistencies, lack of standardized formats, and variations in healthcare terminologies continue to challenge interoperability. Heterogeneous data structures, disparate coding systems, and misalignment between EHR implementations further exacerbate the problem, limiting real-time clinical data exchange and integration. Application Programming Interfaces (APIs) facilitate cross-platform data exchange, yet many healthcare systems struggle with fragmented data repositories and redundant documentation workflows, resulting in data silos that prevent cross-institutional collaboration and continuity of care (21). Established standards such as HL7 FHIR, SNOMED CT, and Clinical Document Architecture (CDA) provide structured data management approaches, but their adoption remains inconsistent across healthcare organizations due to variations in technological maturity, resource constraints, and lack of regulatory mandates (22). Regulatory frameworks such as HIPAA and GDPR enforce compliance measures, but cross-border data-sharing regulations remain fragmented, leading to interoperability bottlenecks in multinational healthcare systems (23).

To address these challenges, standardized technologies such as semantic web frameworks and ontology-based systems, including OWL and RDF, have been proposed to improve interoperability and facilitate more efficient data retrieval and clinical decision-making (24, 25). Despite these advancements, the limited adoption of ontology-based solutions and insufficient alignment between clinical terminologies and ontological models restrict the potential benefits of these technologies. Misalignment between different interoperability models continues to hinder seamless health data exchange, emphasizing the need for further research on developing scalable, domain-specific interoperability solutions that align with evolving clinical and regulatory requirements (26).

### 4.2 Patient-centered care and personalized medicine

PCC emphasizes patient engagement, active participation, and collaborative decision-making, yet existing EHR systems often lack integrated patient-centered tools that support personalized healthcare delivery. Research indicates that structured data models and patient-centric digital platforms enhance engagement, treatment adherence, and health outcomes by providing more accessible, user-friendly patient interfaces tailored to diverse patient populations, including those with limited digital literacy (27). The lack of context-aware digital interventions and intelligent automation in EHR platforms reduces the ability to deliver adaptive, patient-specific recommendations. Designing integrated patient-provider interfaces with digital tools that support self-monitoring and real-time health tracking will be critical in ensuring effective PCC implementation (28). These capabilities are especially crucial in chronic disease management, where ongoing care coordination, patient-reported outcomes, and timely interventions are central to effective treatment. Future frameworks should integrate AI-driven clinical decision support (CDS) systems and real-time patient-reported outcome monitoring to further improve patient engagement and clinical efficiency (29). As shown in Table 3, the reviewed studies highlighted common implementation challenges in PCC and outlined emerging tools to address them.

**Table 3:** Current challenges, gaps, and strategic solutions in patient-centered care and personalized medicine.

Patient-centered care challenge	Gap identified in review	Proposed strategies and tools
Limited integration of PGHD into EHRs.	PGHD is not consistently reflected in clinical workflows; real-time tracking is lacking.	Mobile-first platforms, real-time PGHD syncing, and NLP-based decision-support systems. (27, 28)
Digital health literacy disparities	Older adults, non-tech-savvy users, and underserved populations face barriers to adoption.	Inclusive UX/UI, multilingual interfaces, and user-centered design principles. (30)
Absence of real-time feedback loops.	Lack of context-aware, adaptive interventions limits timely personalization.	AI-enabled CDS tools, automated alerts, and symptom-monitoring apps (29)
Privacy and data control concerns.	Patients lack agency over data access and sharing, reducing trust and participation.	Integration of SSI frameworks and blockchain-based dynamic consent models (31, 32)

### 4.3 Security, compliance, and ethical challenges in genomic data integration

Integrating genomic data into EHRs presents significant privacy, security, and ethical challenges. The high sensitivity of genomic data introduces new risks related to unauthorized access, secondary use of genetic information, and challenges in informed consent management. Concerns over genetic data misuse, privacy violations, and potential discrimination underscore the necessity for robust security protocols to safeguard patient information (33). Compliance with HIPAA (United States) and GDPR (European Union) ensures data protection and regulatory oversight, yet harmonizing these frameworks globally remains a challenge, particularly given the rapid expansion of AI-driven genomic data processing (23). Interoperability gaps in genomic data exchange further exacerbate these challenges, as variations in genetic variant annotations, clinical interpretation models, and data-sharing policies limit the ability to utilize genomic insights effectively.

Genomic data integration also raises concerns about algorithmic bias in AI-driven genetic risk assessments, genetic discrimination, and inequitable access to personalized healthcare services (34). The NIH Ethical Considerations framework emphasizes the importance of transparency in genomic data handling, patient autonomy, and ethical AI governance to mitigate risks associated with genomic data use (35). Studies suggest that achieving a balance between technological advancements and ethical governance is critical for ensuring equitable and responsible genomic data integration in clinical decision-making frameworks (36).

To mitigate risks associated with genomic data integration, future frameworks should incorporate privacy-preserving AI models, federated learning techniques, and blockchain-based consent management to ensure security without compromising data utility (37). Integrating knowledge graphs, such as Neo4j, also represents a promising approach for scalable and secure management of genomic relationships within patient records, improving structured genomic data use in clinical settings (38). The inclusion of Direct Identifiable Information (DII) and Indirect Identifiable Information (III) markers within genomic EHR frameworks could enhance data provenance tracking while minimizing the risk of patient re-identification.

### 4.4 Persistent challenges in current healthcare data integration

This systematic review identifies persistent challenges in healthcare data interoperability, PCC strategies, and genomic data integration. Despite advancements in health IT, semantic inconsistencies, fragmented data architectures, and variations in data exchange protocols continue to hinder seamless interoperability. The findings underscore the need for standardized frameworks that facilitate semantic consistency, secure data exchange, and patient-centric digital tools. Current healthcare systems struggle with real-time interoperability, leading to inefficiencies in data retrieval and clinical workflows. Addressing these limitations requires a multi-faceted approach, including standardizing healthcare terminologies, implementing ontology-driven data models, and enhancing regulatory compliance mechanisms to ensure seamless data exchange across diverse healthcare infrastructures (39). These limitations are especially pronounced in chronic disease management, where fragmented data systems, lack of real-time feedback, and poor integration of longitudinal health data impede continuity of care and personalized treatment.

#### 4.4.1 Genomic data integration and blockchain security

Genomic data integration presents critical challenges related to privacy, security, and ethical concerns, particularly regarding data ownership, consent models, and cross-border genomic data exchange (40). While regulatory frameworks such as HIPAA and GDPR establish foundational compliance measures, global harmonization remains inconsistent, leading to variations in how genomic data is stored, shared, and accessed (41). Ensuring secure and transparent data transactions is essential to mitigate risks associated with genomic data misuse, unauthorized access, and potential genetic discrimination.

One of the emerging solutions to address these security concerns is blockchain technology, which offers decentralized identity management, cryptographic encryption, and transparent access control mechanisms (42). By integrating blockchain-powered SSI models, patients can exercise greater autonomy over their genomic data, allowing them to control access permissions while ensuring tamper-proof audit trails for regulatory compliance (43). Smart contract mechanisms can automate consent management, ensuring that genomic data transactions adhere to evolving regulatory policies (44).

Despite its potential, widespread adoption of blockchain-based solutions in genomic data integration remains limited due to scalability concerns, computational overhead, and interoperability constraints with existing EHR systems (45). Future research should focus on optimizing blockchain frameworks to address scalability limitations, integrating federated learning models for privacy-preserving genomic analysis, and establishing universal standards for blockchain-based genomic data exchange to enhance security and interoperability in precision medicine.

#### 4.4.2 NLP-driven patient engagement

PCC frameworks rely on effective communication, personalized feedback mechanisms, and real-time decision support, yet many digital health platforms lack advanced tools for dynamic patient-provider interactions (42). Natural Language Processing (NLP)-driven models are increasingly being explored to enhance patient engagement by enabling AI-powered chatbots, automated summarization of clinical records, and sentiment analysis of patient-reported outcomes (46).

These technologies facilitate real-time symptom monitoring, adaptive health recommendations, and conversational AI interfaces that improve accessibility for patients with limited digital literacy (44). NLP models can also extract clinically relevant information from unstructured text, enabling automated documentation, enhanced decision support, and improved clinical workflow efficiency (47). Nonetheless, challenges persist in ensuring the accuracy of AI-driven patient responses, reducing bias in NLP models, and integrating these tools into existing EHR systems without introducing additional cognitive load for clinicians (48).

#### 4.4.3 Toward global inclusivity: low- and middle-income countries’ perspectives on digital health

Recent contributions from underrepresented regions reinforce the need to contextualize interoperability, patient-centered care, and genomic data integration within low- and middle-income countries (LMICs). These studies highlight both progress and persistent structural challenges, offering a broader global lens that complements the thematic synthesis presented earlier.

Recent developments in digital health systems from underrepresented regions reinforce the urgency and complexity of achieving true interoperability. A viewpoint analysis of health data integration strategies in India, Kenya, Nigeria, and Indonesia identified FHIR as the preferred standard due to its modular design, open-source availability, and adaptability to LMIC contexts (49). A separate evaluation of eHealth strategies across 34 African countries, based on the World Health Organization–International Telecommunication Union (WHO–ITU) and Findable, Accessible, Interoperable, and Reusable (FAIR) frameworks, revealed significant disparities in interoperability maturity, particularly within national health information exchange infrastructures (50). These studies collectively illustrate the progress made in LMICs as digital health infrastructure continues to evolve in the aftermath of the COVID-19 pandemic, underscoring the importance of technically appropriate standards and regionally responsive governance frameworks.

Recent literature from underrepresented regions also contributes to the evolving conceptualization and implementation of PCC. A 2023 scoping review analyzing PCC definitions across Latin American countries revealed strong alignment with internationally established models, while also highlighting region-specific emphases such as the importance of community involvement and the influence of infrastructural limitations on care delivery (51). In South Africa, a 2024 quantitative survey involving both patients and diagnostic radiographers identified several practical gaps in PCC, including insufficient inclusion of family members during care discussions, limited attention to individualized patient needs, and communication breakdowns between healthcare providers and patients (52). These findings emphasized the importance of embedding culturally grounded values, such as the principle of Ubuntu, into PCC frameworks to support compassionate, context-sensitive care. While PCC principles are widely endorsed, successful adoption in LMICs requires local adaptation to systemic and cultural constraints.

The integration of genomic data into clinical care remains an evolving but essential component of health system transformation in low- and middle-income countries. A 2023 review of personalized medicine efforts across Africa examined several region-led genomic initiatives, including Human Heredity and Health in Africa (H3Africa), African Centre of Excellence for Genomics of Infectious Diseases (ACEGID), and the Southern African Human Genome Programme (SAHGP) (53). These initiatives aim to improve genetic diversity representation, build federated bioinformatics infrastructures, and close the clinical implementation gap. However, barriers such as limited data-sharing capacity, underrepresentation in global datasets, and lack of population-specific genomic references still limit the scalability of integration. This highlights the need for contextual frameworks that enable equitable access to precision medicine across African health systems.

#### 4.4.4 Divergent findings across studies

While the thematic synthesis revealed broad areas of alignment across studies, it also exposed notable contradictions that warrant further attention. Some studies reported successful FHIR-based integration within national health information exchanges, while others highlighted ongoing implementation gaps driven by infrastructural deficiencies and inconsistent semantic mapping. In patient-centered care, although strong conceptual frameworks were common, their translation into practice varied significantly. Some studies emphasized shared decision-making, whereas others reported limited direct patient involvement and a reliance on provider-driven metrics. Similarly, genomic initiatives in Africa such as H3Africa and ACEGID reflect leadership in regional data generation, yet several studies noted challenges in clinical translation due to poor EHR integration or a lack of population-specific genomic references. These divergences underscore the complexity of digital health advancement and reinforce the importance of context-sensitive implementation strategies.

#### 4.4.5 Limitations of existing literature

In addition to the methodological limitations of this review, several recurring weaknesses were noted across the included studies. Many employed small sample sizes or localized populations, limiting the generalizability of findings. Others relied heavily on self-reported data, lacked longitudinal designs, or provided limited methodological transparency. The heterogeneity in study quality and reporting practices also complicated comparative interpretation, particularly across different regions and healthcare contexts. These limitations underscore the need for more robust, multicenter, and implementation-oriented research to inform future digital health integration efforts.

### 4.5 Future directions

Addressing the identified challenges in healthcare data integration requires advancing scalable, secure, and AI-driven interoperability models. While blockchain technology, NLP-driven decision-support tools, and ontology-based frameworks present promising solutions, their integration into existing healthcare ecosystems remains limited (39). Future research must focus on bridging these gaps by optimizing interoperability models, enhancing regulatory alignment, and improving scalability in healthcare IT infrastructures (40).

For genomic data security, blockchain technology has been proposed as a privacy-preserving solution, yet challenges such as scalability limitations, computational overhead, and lack of regulatory harmonization hinder its widespread adoption (44). To overcome these barriers, future efforts should explore lightweight blockchain architectures, federated learning for decentralized data analysis, and interoperability frameworks that enable secure genomic data transactions (54). Strengthening data governance policies will also be critical in ensuring ethical compliance and patient data sovereignty (55).

In PCC, NLP technologies are increasingly applied to support patient engagement through tools such as AI-driven chatbots, automated summaries of clinical interactions, and sentiment analysis of patient-reported data (42). Yet, their practical utility remains limited by persistent issues of data quality, algorithmic bias, and accessibility across diverse populations (56). Future research should explore the integration of ontology-driven clinical decision-support tools in multi-modal datasets, leveraging knowledge graphs and AI reasoning to enhance predictive analytics in precision medicine. Unlike traditional NLP-based tools, ontology-driven systems can infer complex relationships between clinical, genomic, wearable, and EHR data, facilitating adaptive and personalized recommendations (57).

Blockchain security innovations such as zero-knowledge proofs (ZKPs) and multi-party computation (MPC) could further enhance secure data sharing in genomic research while ensuring privacy compliance with GDPR and HIPAA (58). Expanding the role of AI-driven patient engagement through context-aware digital interventions, predictive health analytics, and real-time remote monitoring could revolutionize personalized medicine and support the long-term management of chronic diseases (59).

To ensure long-term impact, a holistic approach is needed—one that aligns AI, blockchain, and ontology-based solutions with standardized healthcare regulations, real-world feasibility studies, and cross-disciplinary collaborations (40). By addressing these technological, ethical, and regulatory gaps, the healthcare industry can transition toward truly integrated, patient-driven, and secure data ecosystems, ultimately enhancing precision medicine, interoperability, and healthcare decision-making (59).

### 4.6 Methodological limitations of this review

While this review followed PRISMA 2020 guidelines and used a structured thematic synthesis approach, several methodological limitations must be acknowledged. First, the search strategy was limited to selected databases including PubMed, MEDLINE, Scopus, WHO, and Google Scholar. Although these sources are comprehensive, others such as Embase, CINAHL, and IEEE Xplore were not included. As a result, relevant literature indexed in those databases may have been missed, potentially narrowing the scope of included studies. Although an English-language filter was applied during the database search, 26 studies were still excluded during the full-text screening phase due to non-English content, as documented in the PRISMA flow diagram. This reflects a common limitation in automated indexing systems, where articles may be retrieved based on English metadata (such as titles or abstracts) despite lacking full English translations. Consequently, the exclusion of non-English full texts may have introduced language bias and limited the global generalizability of findings.

Furthermore, purely quantitative studies and statistical meta-analyses were excluded unless they contributed conceptual insights relevant to our research questions. This may have limited the inclusion of outcome-focused evidence, particularly regarding measurable health improvements. Thematic synthesis was chosen due to the heterogeneity of study designs and outcome measures across the included literature. This approach is well-suited for analyzing qualitative and descriptively reported studies, including those that used mixed-methods designs, and it inherently involves subjective interpretation.

In this review, initial coding was performed manually by identifying key concepts and excerpts from each included study using an inductive approach. Codes were recorded using a structured extraction template and then grouped into broader themes based on recurring patterns across the data. The initial coding was conducted by the first author (RA) and independently reviewed and validated by the second (RSS) and third (JM) reviewers. Discrepancies were resolved through consensus discussions. No statistical inter-rater reliability metric such as Cohen's kappa was calculated, as our approach emphasized conceptual refinement through group consensus rather than quantifying coder agreement.

Risk of bias was assessed using a structured Excel framework that included judgments on study design and reporting transparency. Although this ensured internal consistency, we did not use validated tools such as ROBIS or JBI because our dataset included both qualitative and descriptive implementation studies that did not fully align with the standardized criteria required by those tools. While supplementary sources such as Google Scholar and WHO repositories were included to reduce publication bias, some grey literature and conference abstracts may have offered only partial or preliminary data, which could impact the depth of evidence synthesis. Finally, because most of the included studies were conducted in high-income countries, the findings may not fully apply to underrepresented regions.

## 5 Conclusion

This systematic review underscores the persistent challenges in healthcare data integration, specifically in interoperability, PCC, and genomic data integration. The findings highlight critical gaps in semantic consistency, standardized data-sharing mechanisms, and regulatory alignment, all of which impact the effectiveness of healthcare systems. Despite advancements in health IT frameworks such as HL7 FHIR, SNOMED CT, and ontology-driven data models, interoperability barriers persist due to fragmented architectures, inconsistent adoption of terminologies, and limited cross-platform data exchange capabilities. Addressing these limitations is essential for enhancing clinical workflows, improving data accessibility, and ensuring seamless communication among healthcare providers.

PCC remains central to modern healthcare transformation, yet significant challenges exist in implementing real-time patient feedback mechanisms, NLP-driven engagement tools, and AI-assisted decision support systems. Future healthcare frameworks must integrate dynamic, patient-centric digital tools that facilitate self-monitoring, shared decision-making, and adaptive care models. Genomic data integration presents substantial privacy, security, and ethical concerns, particularly in the context of SSI, consent management, and cross-border genomic data exchange. Blockchain-based governance models and federated learning approaches hold promise for enhancing genomic data security while maintaining regulatory compliance and patient autonomy.

To support both research and practice, this review highlights actionable priorities across three critical domains. Scaling ontology-based interoperability models is essential to ensure alignment with diverse regulatory environments and enable real-time clinical data exchange. Equally important is the integration of culturally resonant PCC strategies into patient-facing technologies, which can enhance real-time feedback, shared decision-making, and coordination in chronic care. At the same time, advancing genomic data security through federated learning and decentralized consent models will help protect privacy while ensuring equitable access to precision medicine. Collectively, these targeted directions provide a foundation for designing integrated, patient-centered, and ethically governed healthcare systems capable of supporting scalable and sustainable digital health infrastructures.

## Data Availability

The datasets presented in this study can be found in online repositories. The names of the repository/repositories and accession number(s) can be found below: https://osf.io/c2xvw/files/osfstorage.

## Supplementary material

Supplementary File S1 PRISMA 2020 Checklist.

Supplementary File S2 Search Strategy.

Supplementary File S3 Study Protocol.

Supplementary File S4 Curated Articles Database (161) (Sheet 1 & 2).

Supplementary File S5 Categorization and Thematic Annotation (Sheet 1 & 2).
