# Supplementary material for: Challenges and strategies in building a foundational digital health data integration ecosystem: a systematic review and thematic synthesis
Source: Front Health Serv. 2025 Jun 20;5:1600689. doi: 10.3389/frhs.2025.1600689 (PMC12226485; doi:10.3389/frhs.2025.1600689)
Supplement: Supplementary File S2 — Search Strategy. [file Datasheet2.pdf]

Foundational Digital Health Data Integration Ecosystem: A Systematic Review and Thematic Synthesis

1. Study Design & Approach

This systematic review follows a qualitative thematic synthesis approach to examine the integration of phenotypic and genotypic data with medical ontology frameworks for enhancing patient-centered care. The methodology was chosen due to the heterogeneity of study designs, interventions, and outcome measures across the included literature.

Given the diverse nature of the included studies, meta-analysis was not feasible. The included studies varied considerably in their methodological approaches, target populations, and reported metrics, which allowed for a structured thematic exploration of key findings.

To ensure clarity and coherence, we adhered to qualitative thematic synthesis principles, systematically categorizing the findings into predefined themes: Interoperability, Patient-Centered Care, and Integration of Genomic Data. This ensured that the synthesis was not only comprehensive but also aligned with the broader research objectives.

The study methodology was developed following PRISMA 2020 guidelines to maintain transparency and reproducibility. A structured PICOS framework was used to guide study selection, ensuring relevance and rigor in data extraction and thematic coding.

By combining a transparent selection process with iterative thematic coding, this study aims to synthesize current evidence on ontology-driven data integration and its relevance to personalized healthcare.

2. Adherence to PRISMA Guidelines

This study follows the PRISMA 2020 guidelines to ensure transparency and rigor in the systematic review process. A PRISMA Flow Diagram has been included to illustrate the study selection

process, detailing the number of records identified, screened, included, and excluded. The diagram is structured to reflect the review stages precisely and aligns with the final study counts.

To enhance clarity, all methodological steps—including study identification, screening, eligibility assessment, and final inclusion—have been mapped explicitly according to PRISMA 2020 standards. This ensures full compliance with systematic review reporting requirements and strengthens the reproducibility of the study.

Additionally, all references to PRISMA compliance are based on PRISMA 2020 guidelines, reinforcing adherence to the latest best practices in systematic review methodology.

3. Literature Search Strategy

The systematic review employed a comprehensive search strategy utilizing Boolean operators to ensure an exhaustive retrieval of relevant literature. Searches were conducted across multiple databases, including PubMed, MEDLINE, Scopus, Google Scholar, and WHO repositories. Additionally, grey literature was included through manual hand-searching to minimize publication bias. To enhance clarity and reproducibility, the following refinements have been incorporated:

- Timeframe for included studies: The search covered studies were identified up to March 15, 2024, ensuring a relevant and up-to-date synthesis of research findings.
- Language restrictions: No language restrictions were applied unless explicitly required for study eligibility, ensuring inclusivity in the review process.
- Search syntax consistency: The search syntax used for PROSPERO registration aligns identically with the final search strategy in this systematic review, ensuring methodological transparency.

These refinements strengthen the rigor and consistency of the literature search process, ensuring comprehensive coverage and alignment with systematic review best practices.

4. Search Syntax Strategy

The search strategy was formulated based on the key concepts outlined in the research objectives, encompassing terms such as "Electronic Health Record," "Computerized Medical Record," "Automated Medical Record," "Clinical Information System," "Health Information System," "Hospital Information System," "Medical Record System," "Genomic data," "Ontology integration," "Chronic conditions," "Patient-centered care," "Patient empowerment," "Interoperability," "Healthcare standards," "Semantic web technologies," "Healthcare quality," "Healthcare delivery," "Patient outcomes," "Data exchange," and "Database design." Boolean operators such as AND, OR, and NOT were used to refine the search terms. Quotation marks ensured phrases were searched as exact terms, while wildcard characters (*) and truncation covered variations in word endings. Parentheses ( ) grouped terms to control the search order. This comprehensive strategy ensured a thorough and systematic search of relevant databases. For both primary databases and supplementary sources, we used a consistent search strategy string. The searches were conducted up to March 15, 2024.

The resulting syntax was ("Electronic Health Record" OR "Computerized Medical Record" OR "Automated Medical Record" OR "Clinical Information System" OR "Health Information System" OR "Hospital Information System" OR "Medical Record System" OR "Genomic data" OR "Ontology integration" OR "Chronic conditions" OR "Patient centered care" OR "Patient empowerment" OR "Healthcare standards" OR "Semantic web technologies" OR "Healthcare quality" OR "Healthcare delivery" OR "Patient outcomes" OR "Data exchange" OR "Database design") AND Interoperability.

5. Article Screening and Selection

Articles potentially relevant to the study were initially screened based on their titles and imported into Rayyan for efficient management and blinded screening by independent reviewers. Further selection involved abstract and full-text reviews, applying Rayyan’s screening tools to eliminate duplicates and filter studies based on predefined inclusion and exclusion criteria in Table 1.

Table 1. Inclusion and Exclusion Criteria

Criteria	Inclusion	Exclusion
Chronic Conditions	Cardiovascular disease, diabetes, asthma, HIV/AIDS, mental health disorders, sensory impairments, musculoskeletal disorders, and oncological conditions	Research not related to chronic conditions or PCC principles
PCC	Focus on patient empowerment, engagement, and active involvement in treatment and health management	Studies not directly related to healthcare information management or PCC principles
EHRs	EHRs, computerized medical records, clinical information systems, health information systems, hospital information systems	Basic science aspects of genomics or ontology without direct relevance to healthcare delivery
Genomic Data Integration	Integrating genomic data for personalized and efficient medical practices	Studies not published in English or lacking full-text availability
Ontology Integration	Improving data interoperability and semantic web technologies in healthcare settings	Letters to the editor, commentary, opinion pieces, or non-original research articles
Healthcare Quality, Delivery, and Outcomes	Research influenced by PCC principles and integrated healthcare frameworks	Research not aligned with improving healthcare quality, patient outcomes and data interoperability
Data Exchange and Interoperability	Data exchange protocols, database design, interoperability standards	Narrative reviews, opinion pieces, or non-original studies
Study Types	Original research findings on healthcare information management	In vitro or animal studies, case reports, meta-analyses, or studies lacking a focus on data integration or PCC.

Study Subjects	Human participants involved in healthcare settings, including clinical workflows and data systems	Studies with healthy controls, age-matched comparisons, single-arm designs, transgenic animals, cadavers, and specific breeds
Sample Types	Studies using human health data relevant to clinical care, patient outcomes, or health information systems	Research involving porcine, fish, soil samples, or cellular analysis

To ensure alignment with study objectives, sample selection prioritized studies involving healthcare providers, clinical workflows, and patient data analysis. Studies that exclusively discussed theoretical ontology models, generic genomic concepts, or computational frameworks without direct application to patient-centered care (PCC), electronic health records (EHRs), or interoperability were excluded.

Given the methodological rigor required for systematic reviews, this study applied a structured selection approach using the PICOS framework (Population, Intervention, Comparator, Outcome, and Study Design). This ensured a transparent, replicable, and focused approach to study selection and research question development, as outlined in Table 2.

Table 2. Research Question Framework (PICOS)

Main Theme	Challenges in Healthcare Data Integration	Clinical Implications	Proposed Solutions & Best Practices
Interoperability	Fragmented data exchange due to lack of standardized protocols<br><br>Inconsistent adoption of HL7 FHIR, SNOMED CT, and LOINC across healthcare systems	Delays in data access and decision-making<br><br>Incomplete or inconsistent patient records<br><br>Limited system scalability	Promote universal adoption of HL7 FHIR, SNOMED CT, and LOINC<br><br>Develop ontology-based models for semantic alignment<br><br>Standardize APIs for seamless platform

			exchange
Patient-Centered Care (PCC)	- Poor integration of PGHD into EHRs<br><br>- Lack of user-friendly tools for patient engagement	- Reduced personalization of care<br><br>- Limited engagement in treatment decisions<br><br>- Lack of real-time feedback	- Develop AI-driven platforms with real-time PGHD tracking<br><br>- Embed CDS tools in EHRs using NLP and feedback loops
Genomic Data Integration	- Ethical and regulatory concerns<br><br>- Consent complexity and cross-border data governance<br><br>- High computational cost of AI/genomics integration	- Data privacy risks and consent violations<br><br>- Barriers to genomic medicine implementation<br><br>- Inaccurate predictions due to processing gaps	- Use federated learning for decentralized genomic analysis<br><br>- Integrate genomic standards (OMOP-CDM, GA4GH) with EHRs<br><br>- Apply privacy-preserving technologies like blockchain and ZKPs

Following these criteria, titles were first assessed for relevance, followed by abstract reviews and a comprehensive review of full-text articles. Only studies meeting the predefined criteria were included, ensuring the integrity and validity of findings. A curated articles database supported this process, see supplementary material

6. Data Extraction and Thematic Categorization

Data extraction was conducted systematically using Rayyan for blinded screening, ensuring unbiased selection and duplicate removal. The extracted data was structured into three predefined themes: Interoperability, Patient-Centered Care (PCC), and Genomic Data Integration, providing a focused framework for thematic synthesis. The PICOS framework was employed to ensure clear alignment between study Population, Intervention, Comparison, Outcomes, and Study design.

To enhance methodological clarity, the following refinements have been incorporated:

- Data extraction process: Two independent reviewers conducted data extraction, with discrepancies resolved through discussion or adjudication by a third reviewer to maintain reliability.
- Data extraction sheet: A standardized data extraction sheet was utilized to systematically record relevant study details. If applicable, supplementary material provides further details on the extraction process.
- PICOS placement: The PICOS framework description is included within the methods section, ensuring appropriate contextualization rather than in the results.

These refinements enhance methodological transparency and data consistency, reinforcing the rigor and reproducibility of the systematic review.

7. Risk of Bias and Quality Assessment

A qualitative risk of bias assessment was conducted to evaluate the methodological rigor and reliability of included studies. Given that this review employs thematic synthesis, which focuses on identifying patterns in qualitative data rather than quantifying study effects, formal risk of bias tools such as ROBIS or Cochrane were deemed inappropriate. Instead, a structured approach was used to assess study quality and potential biases.

To ensure transparency, the following bias categories were evaluated:

- Study design: Appropriateness of study methodology for the research question.
- Reporting transparency: Clarity and completeness in data reporting.
- Funding sources: Identification of potential financial conflicts of interest.
- Conflicts of interest: Declaration of competing interests and researcher affiliations.

For further methodological details on study quality assessment are in Excel. This structured approach ensures a comprehensive evaluation of study quality while maintaining the appropriateness of thematic synthesis in qualitative research.

8. Data Synthesis Approach

Due to the methodological diversity of the included studies, a meta-analysis was not conducted. The heterogeneity in study designs, participant populations, and measured outcomes rendered statistical synthesis infeasible. The studies included qualitative case studies, observational designs, and mixed-methods research, each employing distinct analytical frameworks that did not support uniform effect-size comparisons.

In place of quantitative synthesis, a thematic synthesis approach was adopted to systematically extract recurring patterns, conceptual themes, and research gaps. This method allowed for a structured and transparent integration of findings across diverse study types. To enhance credibility and minimize bias, a manual quality assessment was conducted, evaluating each study's aims, methodology, data collection strategies, analytical rigor, and potential sources of bias. The synthesis process was iterative, involving repeated reviews to ensure consistency and accuracy in theme development.

By integrating principles of systematic review methodology with thematic analysis, this study offers a coherent and reliable synthesis of existing knowledge, supporting the advancement of healthcare data integration frameworks and reinforcing the importance of patient-centered, personalized care.

9. Systematic Coding and Thematic Synthesis

To ensure transparency, rigor, and reliability in our systematic review, a structured thematic synthesis approach was applied to analyze qualitative data. This method allowed for the identification of key themes relevant to healthcare data integration, interoperability, and patient-centered care while maintaining methodological consistency.

9.1 Methodology for Thematic Synthesis

Data Collection and Screening

A comprehensive literature search was conducted across PubMed, MEDLINE, and Scopus, supplemented by Google Scholar and WHO repositories to capture grey literature. The selection process followed PRISMA 2020 guidelines, ensuring transparency in identification, screening, and

eligibility assessment. Articles were screened using Rayyan software to facilitate blinded selection, duplicate removal, and systematic categorization based on predefined inclusion and exclusion criteria.

Initial Coding and Thematic Structuring

The text from selected studies was analyzed using an inductive coding approach to capture key concepts relevant to healthcare data integration. A manual thematic coding process was applied to categorize findings systematically, reducing subjectivity and ensuring alignment with the research objectives. Codes were then refined iteratively to ensure consistency and reproducibility.

Theme Identification and Refinement

The initial codes were systematically grouped into broader thematic categories that reflected common patterns in the data. Through iterative refinement, themes were validated to ensure they accurately represented key challenges and opportunities in healthcare data integration.

9.2 Identified Themes

Thematic synthesis identified three primary themes, each addressing critical challenges in healthcare data interoperability, patient-centered care, and genomic data integration:

1. Interoperability in Healthcare Data Exchange

- Studies highlighted challenges in integrating heterogeneous health data sources, particularly Electronic Health Records (EHRs).
- The adoption of interoperability standards such as HL7 FHIR, along with ontology-driven frameworks, consistently emerged as key solutions across the literature.
- Findings emphasized the importance of standardized APIs and secure cross-platform data integration strategies.

2. Patient-Centered Care and Digital Health Integration

- Studies emphasized how personalized health information enhances patient engagement

and clinical decision-making.

- The integration of digital health tools, including AI-powered decision-support systems, was identified as critical for optimizing patient-provider interactions.
- Findings suggest incorporating patient-generated health data (PGHD) into EHR workflows to improve care personalization.

3. Genomic and Phenotypic Data Integration

- Studies emphasized the growing importance of integrating phenotypic and genotypic data for precision medicine.
- Ethical and security concerns related to genomic data privacy emerged as major barriers to adoption.
- Findings highlighted encryption protocols, decentralized consent models, and federated learning as solutions to enhance secure genomic data sharing.

9.3 Synthesizing Findings for Thematic Analysis

By employing a structured thematic synthesis approach, this study systematically extracted key insights that inform interoperability frameworks, patient-centered care strategies, and genomic data security measures.

8. PRISMA Flow Diagram

The article selection process adhered to the PRISMA 2020 guidelines, providing a transparent and systematic approach to identifying, screening, and including studies in the review. The initial search identified 7,989 records from the specified databases, including supplementary searches from Google Scholar and the WHO repository. After removing duplicates using Rayyan software, 6,148 unique records remained. These were further screened using Rayyan based on predefined inclusion and exclusion criteria, resulting in 1,649 articles assessed for eligibility. Ultimately, 161 articles were included in the systematic review. The PRISMA Flow Diagram (Figure 1) details this process.

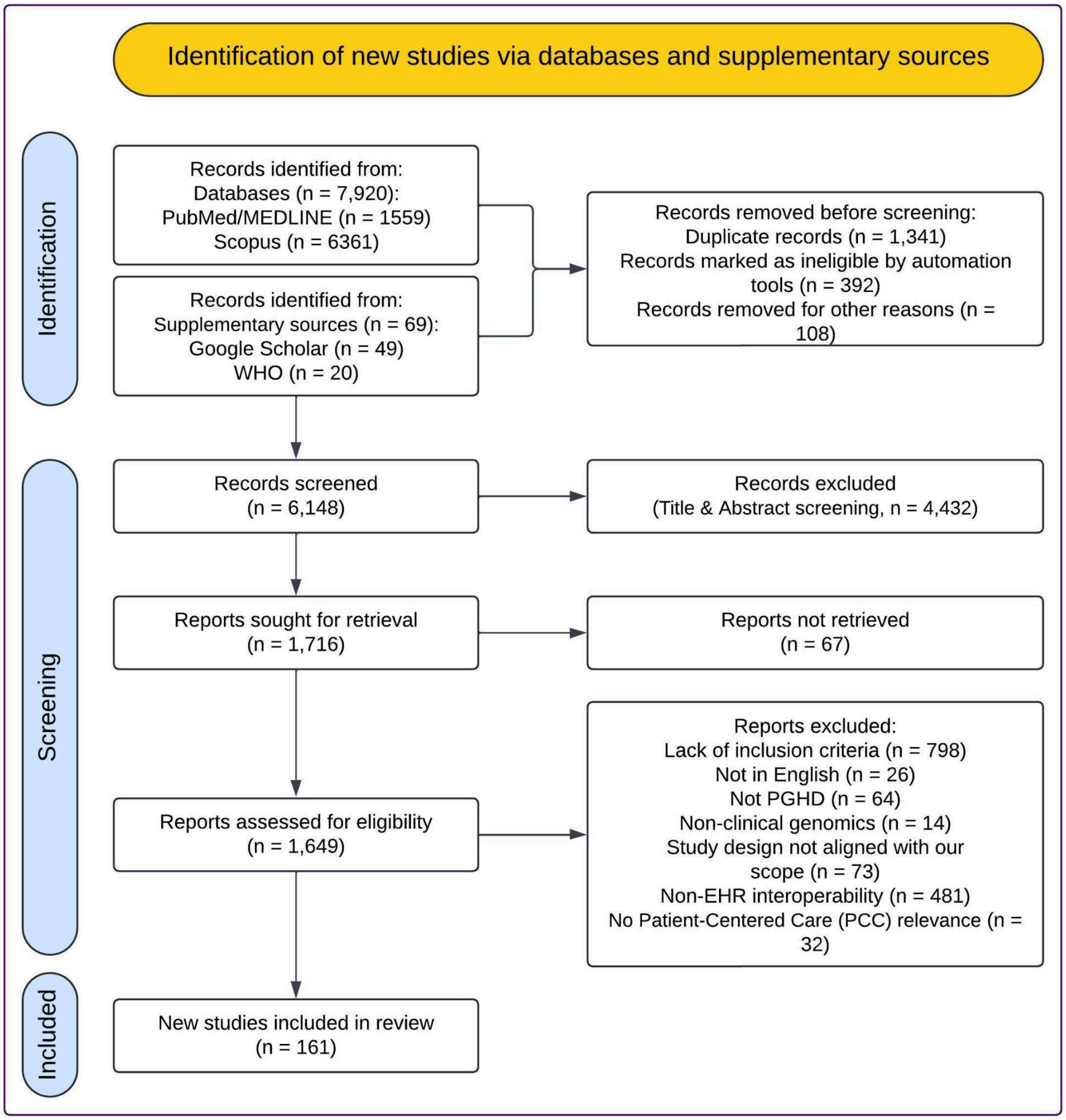

Figure 1. A visual representation illustrating the study selection process according to the Preferred Reporting Items for Systematic Reviews and Meta-Analyses (PRISMA) guidelines. *Note:* PCC, Patient-Centered Care; EHR, Electronic Health Record; PGHD, Patient-Generated Health Data.

8. Key Findings and Research Gaps in Healthcare Data Integration

Thematic synthesis findings were organized into three primary themes: Interoperability, Patient-Centered Care (PCC), and Genomic Data Integration. These themes reflect how existing studies have explored the use of ontology-based technologies, data standards, and patient engagement mechanisms to improve health data integration and delivery.

8.1. Identified Research Gaps

Across the included studies, several recurring gaps were identified:

- **Standardization Challenges**: There is an ongoing need for comprehensive frameworks that ensure seamless integration across diverse and fragmented healthcare systems.
- **Security and Privacy Concerns**: Handling of sensitive data—particularly genomic and phenotypic information—requires stronger safeguards, including encryption and consent management aligned with global standards.
- **PCC Implementation Strategies**: While patient-centered care is widely promoted, limited research investigates how PCC principles can be operationalized within EHRs and clinical workflows.
- **Genomic Data Integration**: Research on the integration of genomic data into healthcare systems remains sparse, especially with regard to ensuring accuracy, scalability, and interpretability.

8.2. Implications for Practice and Future Research

To address these gaps, the literature suggests the following directions:

- Development of scalable interoperability frameworks based on widely adopted standards such as HL7 FHIR, SNOMED CT, and LOINC.
- Implementation of advanced data privacy techniques, including decentralized storage and consent management solutions that comply with GDPR and HIPAA.
- Evaluation of patient-facing tools and interfaces that enhance real-time feedback, data sharing, and engagement.
- Investigation into the use of AI, machine learning, and federated learning to enable ethical, secure, and effective integration of genomic data into clinical decision-making.

By highlighting these thematic insights, the review offers a structured summary of the current state of healthcare data integration and identifies clear pathways for future inquiry and innovation.

Key Findings from Systematic Review and Thematic Synthesis

Main Theme	Challenges in Healthcare Data Integration	Clinical Implications	Proposed Solutions & Best Practices
Interoperability	Fragmented data exchange due to lack of standardized data-sharing protocols. Inconsistent adoption of HL7 FHIR, SNOMED CT, and LOINC across healthcare systems.	Delays in data access, inefficiencies in clinical workflows, and increased risk of medical errors due to incomplete or inaccessible patient records. Limited scalability of health IT systems due to lack of interoperability.	Adoption of HL7 FHIR and ontology-based models to improve semantic consistency. Development of standardized APIs to ensure seamless cross-platform integration. AI-driven terminology mapping tools to address semantic misalignment.
Patient-Centered Care (PCC)	Poor integration of patient-generated health data (PGHD) into EHRs. Lack of user-friendly interfaces for patient engagement.	Reduces personalization of care and affects treatment adherence. Limited engagement in chronic disease management. Lack of real-time feedback delays necessary care adjustments.	Development of AI-enhanced patient portals for real-time monitoring and feedback. Embedding PGHD within EHR workflows to enhance personalized care. Utilizing NLP-based decision-support tools for automated patient guidance.
Genomic Data Integration	Ethical & security concerns around genomic data privacy. Computational inefficiencies in processing large-scale genomic datasets.	Risk of data breaches, regulatory compliance challenges, and barriers to widespread clinical adoption. Incomplete integration of genomic risk factors in decision-making. High computational costs in AI-driven genomic applications.	Implementing secure encryption & decentralized consent models for data privacy. Aligning genomic data standards (OMOP-CDM, GA4GH) with clinical ontologies. Using federated learning techniques to allow AI-driven insights without centralizing data. Developing graph-based databases to support efficient phenotypic-genotypic mapping.
