# Supplementary material for: Challenges and strategies in building a foundational digital health data integration ecosystem: a systematic review and thematic synthesis
Source: Front Health Serv. 2025 Jun 20;5:1600689. doi: 10.3389/frhs.2025.1600689 (PMC12226485; doi:10.3389/frhs.2025.1600689)
Supplement: Supplementary File S3 — Study Protocol. [file Datasheet1.pdf]

Foundational Digital Health Data Integration Ecosystem: A Systematic Review and Thematic Synthesis

The search strategy was developed following PRISMA guidelines and structured around key themes related to healthcare data integration. The key search terms included: "Electronic Health Record," "Computerized Medical Record," "Automated Medical Record," "Clinical Information System," "Health Information System," "Hospital Information System," "Medical Record System," "Genomic data," "Ontology integration," "Chronic conditions,""Patient-centered care," "Patient empowerment," "Interoperability," "Healthcare standards," "Semantic web technologies," "Healthcare quality," "Healthcare delivery," "Patient outcomes," "Data exchange," and "Database design."

The search was conducted in databases such as PubMed/MEDLINE, Scopus, Google Scholar, and the World Health Organization (WHO). To ensure comprehensive coverage, we performed manual searches of reference lists and bibliographies of relevant studies and monitored newly released publications.

The resulting syntax was as follows:

("Electronic Health Record" OR "Computerized Medical Record" OR "Automated Medical Record" OR "Clinical Information System" OR "Health Information System" OR "Hospital Information System" OR "Medical Record System" OR "Genomic data" OR "Ontology integration" OR "Chronic conditions" OR "Patient-centered care" OR "Patient centered care" OR "Patient empowerment" OR "Healthcare standards" OR "Semantic web technologies" OR "Healthcare quality" OR "Healthcare delivery" OR "Patient outcomes" OR "Data exchange" OR "Database design") AND Interoperability

Screening Process:

All retrieved articles were imported into Rayyan for blinded and independent screening by three reviewers. Title screening was followed by abstract and full-text evaluations, applying predefined inclusion and exclusion criteria. Articles were then thematically categorized into three core domains: (1) Interoperability, (2) Patient-Centered Care (PCC), and (3) Genomic

Data Integration. This thematic framework enabled the structured extraction of challenges, opportunities, and proposed solutions related to digital health data integration.

Study Selection:

The PICOS framework (Population, Intervention, Comparator, Outcome, and Study Design) was used to guide initial eligibility criteria. However, final study selection was driven by thematic synthesis. All discrepancies between reviewers were resolved through discussion and consensus.

This systematic review was prospectively registered with the Open Science Framework (OSF): https://osf.io/c2xvw

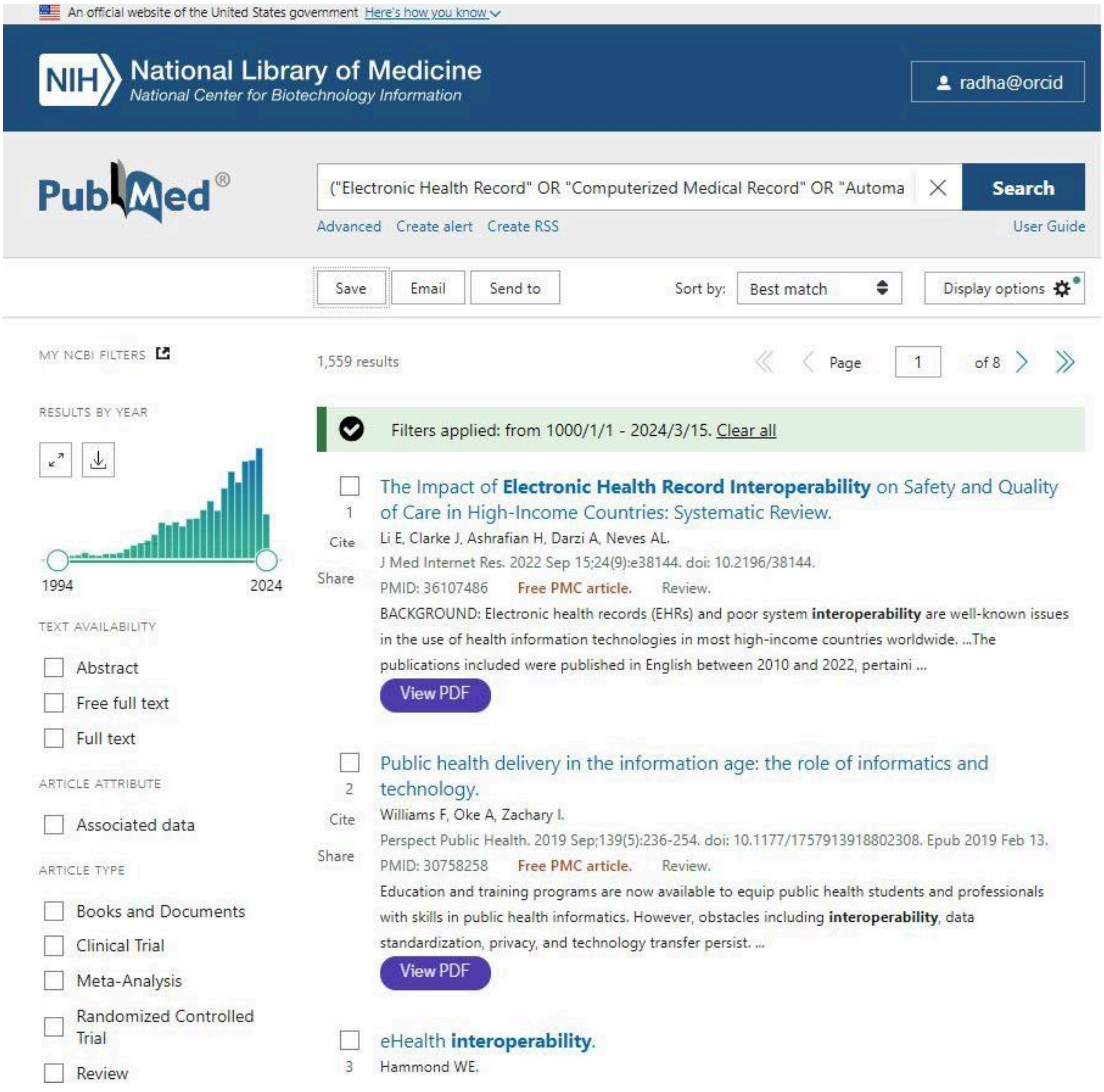

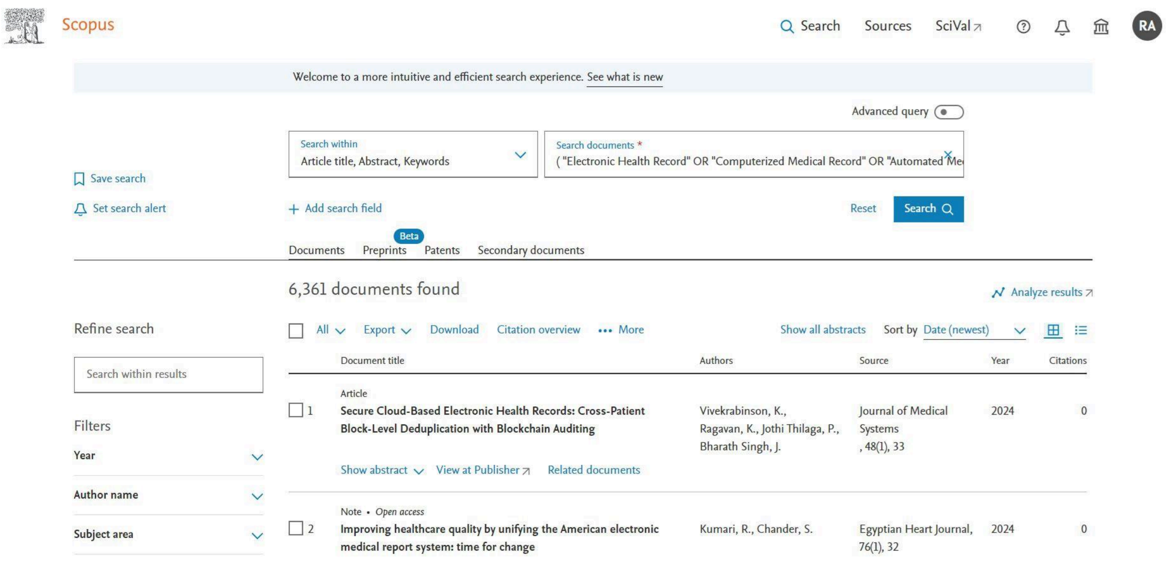
Scopus
Search
Sources
SciVal
RA
Welcome to a more intuitive and efficient search experience. See what is new
Advanced query
Save search
Set search alert
Search within
Article title, Abstract, Keywords
Search documents *
("Electronic Health Record" OR "Computerized Medical Record" OR "Automated Me
Add search field
Reset
Search
Documents
Preprints
Beta
Patents
Secondary documents
6,361 documents found
Analyze results
Refine search
Search within results
All
Export
Download
Citation overview
More
Show all abstracts
Sort by
Date (newest)
Document title
Authors
Source
Year
Citations
Filters
Year
Author name
Subject area
Article
1
Secure Cloud-Based Electronic Health Records: Cross-Patient Block-Level Deduplication with Blockchain Auditing
Vivekrabinson, K., Ragavan, K., Jothi Thilaga, P., Bharath Singh, J.
Journal of Medical Systems , 48(1), 33
2024
0
Show abstract
View at Publisher
Related documents
Note • Open access
2
Improving healthcare quality by unifying the American electronic medical report system: time for change
Kumari, R., Chander, S.
Egyptian Heart Journal, 76(1), 32
2024
0

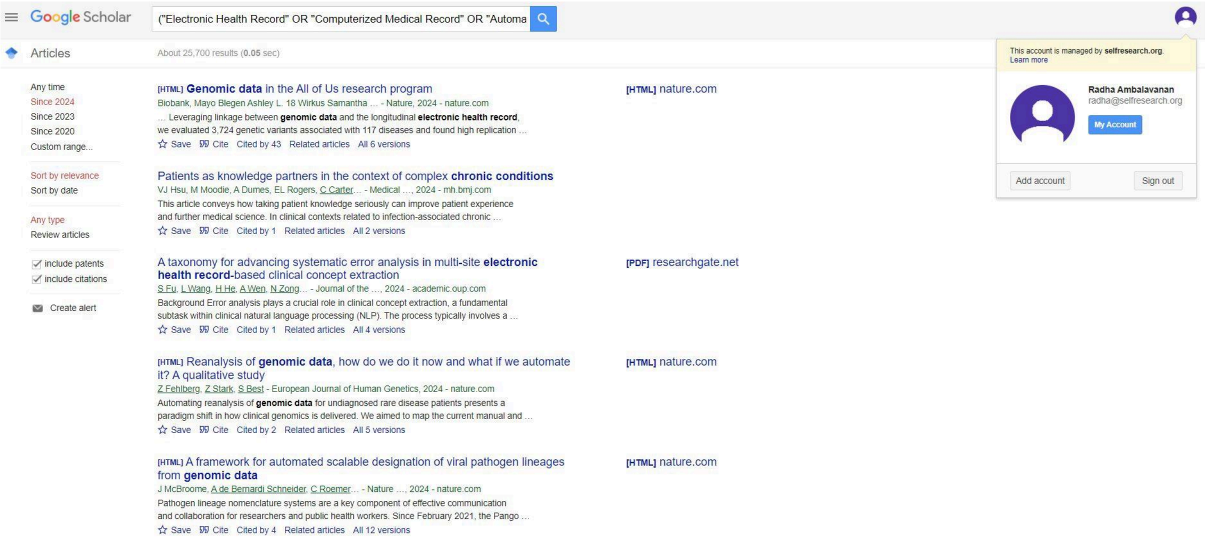
Google Scholar
("Electronic Health Record" OR "Computerized Medical Record" OR "Automa
Articles
About 25,700 results (0.05 sec)
Any time
Since 2024
Since 2023
Since 2020
Custom range...
Sort by relevance
Sort by date
Any type
Review articles
include patents
include citations
Create alert
[HTML] Genomic data in the All of Us research program
Biobank, Mayo Blegen Ashley L. 18 Wirkus Samantha ... - Nature, 2024 - nature.com
... Leveraging linkage between genomic data and the longitudinal electronic health record, we evaluated 3,724 genetic variants associated with 117 diseases and found high replication ...
Save Cite Cited by 43 Related articles All 6 versions
[HTML] nature.com
Patients as knowledge partners in the context of complex chronic conditions
VJ Hsu, M Moodie, A Dumes, EL Rogers, C Carter... - Medical ..., 2024 - mh.bmj.com
This article conveys how taking patient knowledge seriously can improve patient experience and further medical science. In clinical contexts related to infection-associated chronic ...
Save Cite Cited by 1 Related articles All 2 versions
A taxonomy for advancing systematic error analysis in multi-site electronic health record-based clinical concept extraction
S Fu, L Wang, H He, A Wen, N Zong... - Journal of the ..., 2024 - academic.oup.com
Background Error analysis plays a crucial role in clinical concept extraction, a fundamental subtask within clinical natural language processing (NLP). The process typically involves a ...
Save Cite Cited by 1 Related articles All 4 versions
[PDF] researchgate.net
[HTML] Reanalysis of genomic data, how do we do it now and what if we automate it? A qualitative study
Z Fehlberg, Z Stark, S Best - European Journal of Human Genetics, 2024 - nature.com
Automating reanalysis of genomic data for undiagnosed rare disease patients presents a paradigm shift in how clinical genomics is delivered. We aimed to map the current manual and ...
Save Cite Cited by 2 Related articles All 5 versions
[HTML] nature.com
[HTML] A framework for automated scalable designation of viral pathogen lineages from genomic data
J McBroome, A de Bernardi Schneider, C Roemer... - Nature ..., 2024 - nature.com
Pathogen lineage nomenclature systems are a key component of effective communication and collaboration for researchers and public health workers. Since February 2021, the Pango ...
Save Cite Cited by 4 Related articles All 12 versions
[HTML] nature.com
This account is managed by selfresearch.org.
Learn more
Radha Ambalavanan
radha@selfresearch.org
My Account
Add account
Sign out

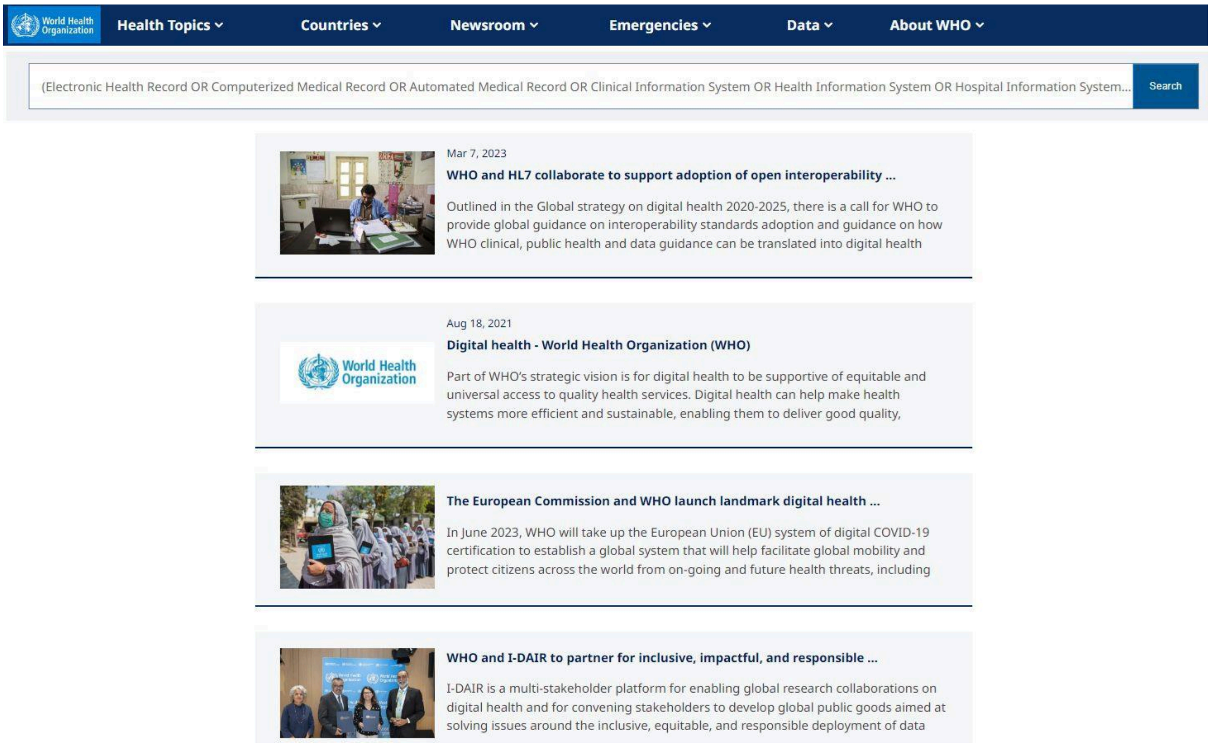
World Health Organization
Health Topics
Countries
Newsroom
Emergencies
Data
About WHO
(Electronic Health Record OR Computerized Medical Record OR Automated Medical Record OR Clinical Information System OR Health Information System OR Hospital Information System...
Search
Mar 7, 2023
WHO and HL7 collaborate to support adoption of open interoperability ...
Outlined in the Global strategy on digital health 2020-2025, there is a call for WHO to provide global guidance on interoperability standards adoption and guidance on how WHO clinical, public health and data guidance can be translated into digital health
Aug 18, 2021
Digital health - World Health Organization (WHO)
Part of WHO's strategic vision is for digital health to be supportive of equitable and universal access to quality health services. Digital health can help make health systems more efficient and sustainable, enabling them to deliver good quality,
The European Commission and WHO launch landmark digital health ...
In June 2023, WHO will take up the European Union (EU) system of digital COVID-19 certification to establish a global system that will help facilitate global mobility and protect citizens across the world from on-going and future health threats, including
WHO and I-DAIR to partner for inclusive, impactful, and responsible ...
I-DAIR is a multi-stakeholder platform for enabling global research collaborations on digital health and for convening global public goods aimed at solving issues around the inclusive, equitable, and responsible deployment of data

----
